# Laparoscopic transabdominal preperitoneal obturator hernioplasty with self-gripping mesh: A case report with operative video

**DOI:** 10.1016/j.ijscr.2021.106657

**Published:** 2021-12-05

**Authors:** Kenta Doden, Takahiro Yoshimura, Yoshitaka Iwaki, Hideaki Kato, Masahiko Kawaguchi, Toru Watanabe

**Affiliations:** Department of Surgery, Yokohama Sakae Kyosai Hospital, 132 Katsura-cho, Yokohama 247-8581, Japan

**Keywords:** CT, computed tomography, CRP, C-reactive protein, OH, obturator hernia, POD, post-operative day, TAPP, transabdominal preperitoneal, TEP, totally extraperitoneal, WBC, white blood cell, Obturator hernia, Self-gripping mesh, TAPP

## Abstract

**Introduction:**

We investigated the effectiveness of a self-gripping mesh, which has microgrips attached to fibrous tissue, in laparoscopic transabdominal preperitoneal (TAPP) obturator hernia (OH) repair to minimize the risk of postoperative pain and obturator nerve injury.

**Presentation of case:**

The patient was an 80-year-old woman who was transferred to our emergency department with abdominal pain in the right lower quadrant and low back pain that began half a day prior to presentation. Computed tomography (CT) detected right OH. Based on the results of the laboratory examination and dynamic CT, intestinal viability was maintained. Ultrasonography-assisted manual reduction of the incarcerated intestine was performed, followed by admission to our department to check for delayed perforation of the intestine. Laparoscopic TAPP OH repair was performed on day seven as an elective surgery. A self-gripping mesh was placed over the OH defect and the femoral ring without tacking. The patient was discharged on postoperative day four, without any complications.

**Discussion:**

Tacking of the mesh at the lateral and dorsal sides of the obturator canal is dangerous due to the presence of the obturator nerve and vessels. Self-gripping mesh use in laparoscopic TAPP OH repair is a rational decision in terms of avoiding tacking or suturing around the obturator canal while maintaining stable fixation of the mesh to prevent recurrence.

**Conclusion:**

Laparoscopic TAPP OH repair with self-gripping mesh is a rational treatment option that reduces the risk of obturator nerve injury while maintaining the secure fixation of a mesh to prevent recurrence.

## Highlights

• Laparoscopic obturator hernia repair with a self-gripping mesh gives successful results.
• Advantages include the lack of need for tacking the mesh and ensuring stable fixation.
• This is a rational option for obturator hernia repair.

## 1 Introduction

Obturator hernia (OH) is a rare type of pelvic hernia that can cause bowel obstruction and often requires emergency surgery. Although the obturator nerve is close to the obturator defect, its observation is difficult during laparotomy because of its deep operative field. Recently, laparoscopic transabdominal transperitoneal (TAPP) OH repair has been widely performed, and its advantages over the open approach have been reported [1]. Laparoscopic observation of the obturator canal is better than that of laparotomy, and the laparoscopic approach reduces morbidity and postoperative hospital stay [1]. However, inappropriate mesh placement may cause severe pain in the groin and medial thigh areas and walking difficulty [2]. In other words, the type of mesh and its fixing technique are important to repair OH without complications.

Self-gripping mesh, which has microgrips that attach quickly and easily to fibrous tissue, enables tack-free fixation [3]. Therefore, we investigated the effectiveness of this tack-free mesh in laparoscopic TAPP OH repair to minimize the risk of postoperative pain and obturator nerve injury. This study follows the surgical case report (SCARE) guidelines [4].

## 2 Presentation of case

**Fig. 1:**
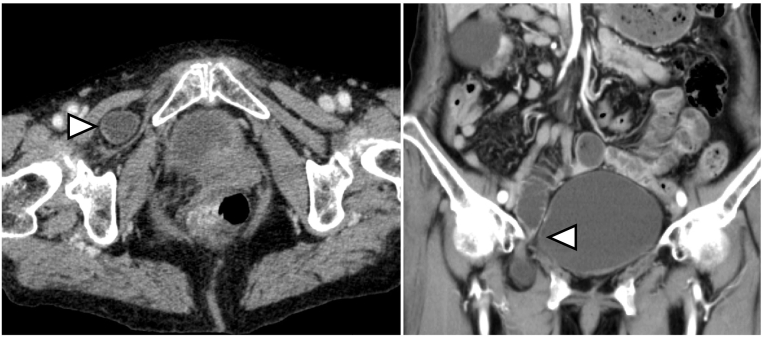
Computed tomography detected right obturator hernia. The oral side of strangulated intestine was slightly dilated.

An 80-year-old woman with a medical history of appendectomy for appendicitis, hypertension, and cerebral infarction was transferred to our emergency department for abdominal pain in the right lower quadrant and low back pain that began half a day prior to presentation. Laboratory evaluation detected increased white blood cell counts (12,800/μL), while other tests, including C-reactive protein (CRP, 0.13 mg/dL) and blood gas analysis, were normal. Computed tomography (CT) revealed a right obturator hernia. The oral side of the strangulated intestine was slightly dilated (Fig. 1). Dynamic CT revealed a sufficient contrast effect of the intestine. Based on these results, we hypothesized that the intestinal viability was maintained. Therefore, manual reduction of the incarcerated hernia was performed using ultrasonography. She was admitted to our department and was followed up to check for delayed perforation. A day after admission, laboratory examination and CT were performed again, and oral intake was started. Then, we planned TAPP OH repair on day seven as an elective surgery (see Video 1).

**Fig. 2:**
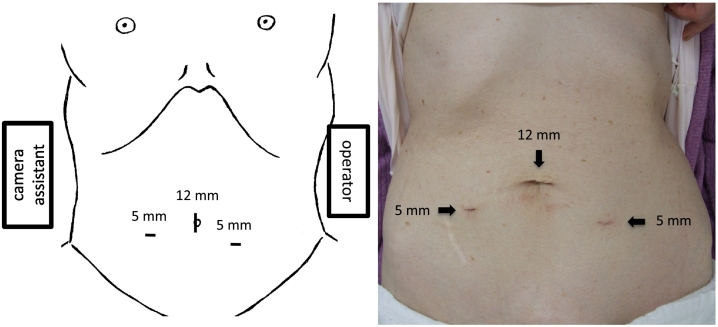
A 12-mm trocar for the endoscope at umbilicus and two 5-mm trocars for the surgeon were inserted.

**Fig. 3:**
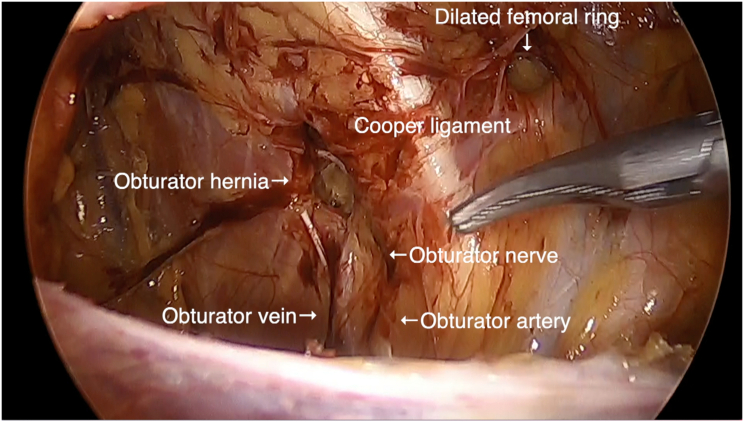
Tacking of mesh at lateral and dorsal side of the canal is dangerous due to the presence of obturator nerve and vessels.

**Fig. 4:**
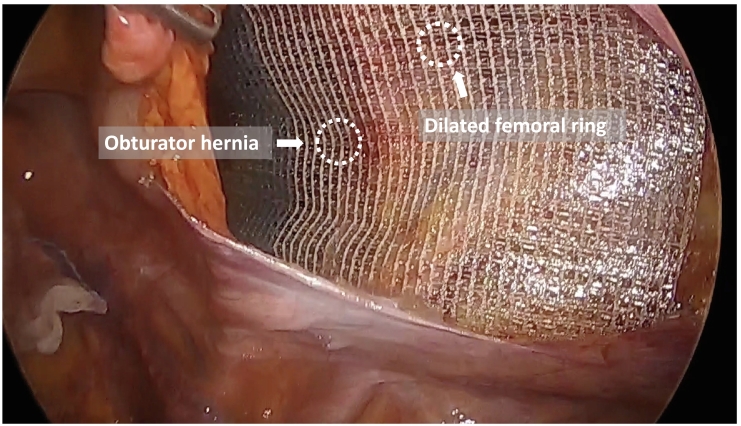
Self-gripping mesh was placed over the obturator hernia defect and femoral ring without tacking.

Under general anesthesia, the patient was placed in a supine position. The surgeon stood on the left side of the patient, and the camera assistant stood on the right side of the patient. Fig. 2 shows the trocar positioning during the operation. A 12-mm trocar for the endoscope at the umbilicus and two 5-mm trocars for the surgeon were inserted. The small bowel was carefully moved away from the operative field by changing the patient's position to the Trendelenburg position. Hernia defects were observed around the right obturator canal. An occult obturator hernia was not observed on the left side. Incarcerated intestines that were reduced seven days prior to the operation were not distinguished. Then, a peritoneal incision was made 1 cm below the internal inguinal ring, and dissection of the preperitoneal space was started. A dilated femoral ring was observed in addition to an obturator hernia (Fig. 3). The obturator hernia sac was carefully reduced. The round ligament of the uterus was ligated using ultrasonic shears. Measuring around the obturator hernia and femoral ring, self-gripping mesh (ProGrip™, Medtronic Inc., Dublin, Ireland) was cut to 7 cm × 10 cm, which was sufficient to cover both OH defects and the femoral ring. The mesh was inserted through the 12-mm trocar towards the lateral preperitoneal space, and it was placed over the OH defect and femoral ring (Fig. 4). The edge of the mesh was unfolded and gently pressed into the tissue. Tacking was not performed. The peritoneum was completely closed with absorbable running sutures. The operation time was 62 min, and intraoperative blood loss was 1 mL. Oral intake was started on postoperative day (POD) 1, and oral antiplatelet therapy was started on POD 2. The patient was discharged on postoperative day 4 without any complications. Six months after the operation, no signs of recurrence or chronic pain were observed.

## 3 Discussion

We report from our experience with this patient that laparoscopic TAPP OH repair with self-gripping mesh is a suitable procedure that reduces the risk of obturator nerve injury. TAPP OH repair with self-gripping mesh is not well reported; this may be because OH is a rare type of hernia. On the other hand, the self-gripping mesh is widely used for laparoscopic TAPP inguinal hernia repair. The use of self-gripping mesh has been reported to reduce chronic pain in laparoscopic TAPP inguinal hernia repair [5], while the use of self-gripping mesh in open inguinal hernia repair does not decrease the incidence of chronic pain [3], [6], [7]. This may be because the self-gripping mesh reduces the risk of postoperative pain related to excessive tack use in TAPP inguinal hernia repair [8]. As for obturator hernia, the risk of mesh fixation with tacking or suture is higher than that of inguinal hernia. Although the obturator canal should be covered with mesh sufficiently to prevent recurrence, tacking of the mesh at the lateral and dorsal sides of the canal is dangerous due to the presence of the obturator nerve and vessels (Fig. 3). The tacking of a flat mesh on the nerve will result in severe postoperative complications, such as severe pain in the groin and medial thigh areas and walking difficulty [2]. Insertion of the plug type mesh to the obturator canal may also cause compression of the nerve. Petrushnko et al. reported that self-gripping mesh use in totally extraperitoneal OH repair was useful in avoiding fixation around the obturator canal [9]. We report from this experience that self-gripping mesh is a rational procedure to reduce the risk of obturator nerve injury for laparoscopic TAPP OH repair.

Self-gripping mesh use may be difficult to use in an emergency setting. Bowel obstruction and intestinal distension make it difficult to secure the operative field to dissect the preperitoneal space and properly place the self-gripping mesh [10]. In the case of TAPP following manual reduction [10] or two-stage TAPP [11], the operative field is secured, and the use of a self-gripping mesh is suitable. For manual reduction of OH, predicting poor bowel viability, which requires bowel resection, is necessary. Kawanaka et al. reported that among patients who underwent small bowel resection, days from symptom onset to operation were significantly longer and preoperative white blood cell (WBC) counts and CRP levels were significantly higher [10]. The receiver operating characteristic curves for predicting poor bowel viability and the optimal cutoff levels for symptom onset to operation, WBC counts, and CRP levels were 2 days, 6530/mL, and 1.20 mg/dL, respectively. Since there are no criteria to proceed with manual reduction of OH, this prediction may be applicable to reduction. In our case, the duration of symptom onset to presentation was half a day, and the CRP level was 0.13, which is lower than the reported cutoff levels. Although WBC counts were higher than the cutoff levels, other examinations including dynamic CT and blood gas analysis indicated bowel viability. Thus, we performed a manual reduction of the incarcerated OH.

Since only one surgical case was presented without a learning curve, this was a major limitation of this report. Large-scale prospective studies are required to confirm the effectiveness of the self-gripping mesh in TAPP OH repair as this reduces the risk of both obturator nerve injury and recurrence.

## 4 Conclusion

Laparoscopic TAPP OH repair with self-gripping mesh is a suitable procedure that reduces the risk of obturator nerve injury while maintaining the stable fixation of the mesh to prevent recurrence.

The following is the supplementary data related to this article.

Video 1 Laparoscopic transabdominal preperitoneal obturator hernia repair with self-gripping mesh. Video 1

## Funding

The authors declare that they received no funding support for this report.

## Ethical approval

The need for ethical approval for this report was waived by the Ethics Committee of the by our institution.

## Consent

Written informed consent was obtained from the patient for publication of this case report and accompanying images. A copy of the written consent is available for review by the Editor-in-Chief of this journal on request.

## Availability of data and materials

The datasets supporting the conclusions of this article are included within the article.

## Author contributions

K.D. prepared the original draft. K.D. and T.Y. performed the experiments. K.D. prepared the original draft. Other authors contributed to perioperative management and data collection. All authors have read and agreed to the published version of the manuscript.

## Registration of research studies

Not applicable.

## Guarantor

Kenta Doden, corresponding author of this article.

## Declaration of competing interest

The authors have no competing interests to disclose.

## References

[1] Schizas D., Apostolou K., Hasemaki N., Kanavidis P., Tsapralis D., Garmpis N. (2021). Obturator hernias: a systematic review of the literature. Hernia.

[2] Haninec P., Horak L., Kaiser R. (2013). Obturator nerve injury in laparoscopic inguinal hernia mesh repair. Hernia.

[3] Molegraaf M., Kaufmann R., Lange J. (2018). Comparison of self-gripping mesh and sutured mesh in open inguinal hernia repair: a meta-analysis of long-term results. Surgery.

[4] Agha R.A., Franchi T., Sohrabi C., Mathew G., Kerwan A., SCARE Group (2020). The SCARE 2020 guideline: updating consensus Surgical Case Report (SCARE) guidelines. Int. J. Surg..

[5] Fumagalli Romario U., Puccetti F., Elmore U., Massaron S., Rosati R. (2013). Self-gripping mesh versus staple fixation in laparoscopic inguinal hernia repair: a prospective comparison. Surg. Endosc..

[6] Li J., Ji Z., Li Y. (2014). The comparison of self-gripping mesh and sutured mesh in open inguinal hernia repair: the results of meta-analysis. Ann. Surg..

[7] Axman E., Holmberg H., Nordin P., Nilsson H. (2020). Chronic pain and risk for reoperation for recurrence after inguinal hernia repair using self-gripping mesh. Surgery.

[8] Belyansky I., Tsirline V.B., Klima D.A., Walters A.L., Lincourt A.E., Heniford T.B. (2011). Prospective, comparative study of postoperative quality of life in TEP, TAPP, and modified lichtenstein repairs. Ann. Surg..

[9] Petrushnko W., Isaacs A., Hackland T., Ghusn M. (2019). Case report: laparoscopic totally extraperitoneal repair of an obturator hernia with self-gripping mesh under spinal anaesthesia. Int. J. Surg. Case Rep..

[10] Kawanaka H., Hiroshige S., Kubo N., Hirashita T., Masuda T., Kaisyakuji Y. (2018). Therapeutic strategy for incarcerated obturator hernia using preoperative manual reduction and laparoscopic repair. J. Am. Coll. Surg..

[11] Sasaki A., Takeuchi Y., Izumi K., Morimoto A., Inomata M., Kitano S. (2016). Two-stage laparoscopic treatment for strangulated inguinal, femoral and obturator hernias: totally extraperitoneal repair followed by intestinal resection assisted by intraperitoneal laparoscopic exploration. Hernia.

